# Editorial: Cross-sectoral collaboration in inclusive education

**DOI:** 10.3389/fpsyg.2025.1697199

**Published:** 2025-10-01

**Authors:** Jie Zhang, Vera Capellini, Eduardo Pimentel da Rocha, Dionísia Aparecida Cusin Lamônica, Yolanda Muñoz-Martinez

**Affiliations:** ^1^Department of Education, Languages, and Instructional Design, State University of New York (SUNY) Brockport, Brockport, NY, United States; ^2^Department of Education, São Paulo State University, Bauru, Brazil; ^3^Department of Speech-Language Pathology and Audiology, University of São Paulo, São Paulo, Brazil; ^4^Department of Educational Sciences, University of Alcalá, Alcalá de Henares, Spain

**Keywords:** inclusive education, cross-sectoral collaboration, educational policy, mental health support, educators, teacher preparation, family engagement

## Introduction

Research demonstrates that intersectoral collaboration transforms educational practices, fostering inclusive cultures, strengthening community partnerships, and advancing institutional sustainability (Maier and Niebuhr, 2021; Kania et al., 2021). The United Nations Educational, Scientific and Cultural Organization (UNESCO) reports that educational outcomes are strongly correlated with factors such as physical and mental health and socioeconomic status (United Nations Educational, Scientific and Cultural Organization, 2020), emphasizing the need for integrated services through cross-sectoral collaboration to address the diverse needs of all learners (United Nations Educational, Scientific and Cultural Organization, 2020). This Research Topic, “*Cross-sectoral collaboration in inclusive education*,” edited by Capellini, Zhang, Lamônica, Muñoz-Martinez, and Rocha for *Frontiers in Psychology*, expands the existing literature by providing theoretical and practical studies on how intersectoral partnerships can effectively address learners' diverse needs while fostering inclusive educational environments worldwide.

## Multi-professional collaboration and support systems

This Research Topic directly responds to United Nations Educational, Scientific and Cultural Organization's (2020) call for collaboration across sectors, investigating how integrated service delivery can enhance service quality and optimize cost-efficiency in meeting diverse learners' needs. It also examines the mechanisms of how interprofessional partnerships can advance inclusive education.

Christensen's study of Norwegian kindergarten teachers recognizes education's crucial role in mental health and psychosocial support through establishing routines, building relationships, and creating safe learning environments. Their work challenges traditional intervention models and positions education as an essential component within comprehensive, community-collaborative support systems.

Vidal et al.'s conceptual analysis demonstrates how systematic collaboration across education, health, and policy sectors in Chile fosters inclusive cultures for students with disabilities, indigenous populations, and socioeconomically disadvantaged groups. Their model proposes actionable guidelines that overcome structural barriers and integrate inclusive practices in schools, addressing diverse student needs through coordinated sectoral support.

Tokheim et al. examine how Norwegian alternative educational programs re-engage students aged 9–16 who are at risk of school dropout by empowering them to make informed decisions about their learning and wellbeing. Their research demonstrates how alternative programs enhance students' self-esteem and autonomy through prioritizing student agency and providing individualized support.

Chahboun et al. investigate how Norwegian schools identify and support students with developmental language disorders through accessible tools, targeted initiatives, and balanced pedagogical practices. Their study emphasizes the importance of interprofessional collaboration among all professionals to create well-structured, inclusive environments that promote academic success and social integration for students with language disorders.

## Cultural diversity and linguistic inclusion

Aligning with United Nations Educational, Scientific and Cultural Organization's (2020) broad definition of inclusion, this Research Topic addresses multicultural education and sign language inclusion. The papers demonstrate how different pedagogical approaches can effectively serve diverse learner populations in various cultural contexts.

Kalogerogianni demonstrates how intercultural pedagogy encourages dialogue between cultures to overcome monocultural paradigms, build democratic global citizenship, and foster respect for cultural diversity in multicultural classrooms. The study advocates for integrating intercultural objectives, methodologies, and digital tools across curricula to enhance language acquisition while preparing students to participate in contemporary multicultural societies.

Morales Acosta and Lattapiat Navarro analyze Chilean Sign Language translations by two Deaf co-teachers, revealing the sophisticated pedagogical strategies educators adapt to preserve and transmit cultural knowledge while ensuring accessibility for Deaf students. The investigation demonstrates how Deaf students are involved as active agents in the translation process, incorporating linguistic and cultural aspects of the Deaf community.

## Meaningful participation of stakeholders: perspectives from students, professionals, and families

Following United Nations Educational, Scientific and Cultural Organization (2020)'s call for dialogue, openness, and participation, this Research Topic incorporates perspectives from professionals, students, and families, ensuring those affected by inclusive education policies and practices have a voice. The diverse national contexts represented—Brazil, China, Chile, and Norway—reflect United Nations Educational, Scientific and Cultural Organization (2020)'s recognition that inclusive education must adapt to local circumstances while maintaining universal principles.

Capellini, Muñoz-Martínez et al.'s study, “*Students' perception of the strengths and weaknesses of the inclusive educational model of Brazilian municipal public schools*,” investigates how 6- and 7-year-old children perceive inclusive education in municipal schools. Results indicate that children identify infrastructural and urban barriers as weaknesses, while recognizing difference-embracing practices and positive relationships as strengths that foster inclusivity. The study reinforces the importance of promoting inclusion from the earliest school years by respecting differences and embracing inclusivity.

Another study led by Capellini, Zhang et al., “*Positive and negative aspects of remote learning during the COVID-19 pandemic in the view of Brazilian university students who received university assistance*,” examines how Brazilian undergraduate students perceived remote learning, focusing on institutional support systems and technological adaptations for educational continuity. This research provides evidence on how support mechanisms enhance student retention and promote inclusive access to higher education during times of crisis.

Huang investigates the impact of empathy and teacher efficacy on the relationship between mindfulness and inclusive attitudes among elementary school teachers. The work highlights the importance of preparing educators for cross-sectoral collaboration, provides theoretical foundations to understand psychological mechanisms, and offers practical guidance for schools to implement mindfulness-based interventions for educators.

Tebar-Yébana et al. conducted a bibliometric analysis of 77 papers across 39 nations, revealing growing recognition of parental perspectives on raising children with intellectual disabilities within inclusive education frameworks. Their study suggests that family satisfaction is linked to the success of educational inclusion. It demonstrates how diverse community contexts significantly shape the implementation and effectiveness of inclusive educational practices globally.

## Future directions, challenges, and conclusion

This Research Topic presents research evidence that cross-sectoral collaboration is fundamental to cultivating inclusive learning environments. While studies demonstrate potential for collaboration among education, health, social services, and policy sectors, they also illustrate the complexity of intersectoral partnership in advancing inclusive education internationally. The Research Topic reveals structural barriers and systemic inequities that often impede effective collaboration. Future research must develop sustainable inter-agency cooperation models and address systemic inequities faced by students from marginalized communities across cultures, languages, and socioeconomics in various geographic contexts.

We extend our gratitude to all authors and expert reviewers. Their rigorous work advances our understanding of building more inclusive educational communities through meaningful cross-sectoral collaboration across nations.
